# Regulation of Hedgehog Signaling by miRNAs and Nanoformulations: A Possible Therapeutic Solution for Colorectal Cancer

**DOI:** 10.3389/fonc.2020.607607

**Published:** 2021-01-07

**Authors:** Zeeshan Javed, Muhammad Javed Iqbal, Amna Rasheed, Haleema Sadia, Shahid Raza, Asma Irshad, Wojciech Koch, Wirginia Kukula-Koch, Anna Głowniak-Lipa, William C. Cho, Javad Sharifi-Rad

**Affiliations:** ^1^ Office for Research Innovation and Commercialization, Lahore Garrison University, Lahore, Pakistan; ^2^ Department of Biotechnology, Faculty of Sciences, University of Sialkot, Sialkot, Pakistan; ^3^ School of Basic Medical Sciences, Lanzhou University, Lanzhou, China; ^4^ Department of Biotechnology, Balochistan University of Information Technology, Engineering and Management Sciences, Quetta, Pakistan; ^5^ Department of Life Sciences, University of Management and Technology, Lahore, Pakistan; ^6^ Chair and Department of Food and Nutrition, Medical University of Lublin, Lublin, Poland; ^7^ Department of Pharmacognosy, Medical University of Lublin, Lublin, Poland; ^8^ Department of Cosmetology, University of Information Technology and Management in Rzeszów, Rzeszów, Poland; ^9^ Department of Clinical Oncology, Queen Elizabeth Hospital, Kowloon, Hong Kong; ^10^ Phytochemistry Research Center, Shahid Beheshti University of Medical Sciences, Tehran, Iran; ^11^ Facultad de Medicina, Universidad del Azuay, Cuenca, Ecuador

**Keywords:** Hedgehog signaling, miRNAs, nanoformulations, nanoparticle, therapeutics, colorectal cancer

## Abstract

Hedgehog (Hh) signaling aberrations trigger differentiation and proliferation in colorectal cancer (CRC). However, the current approaches which inhibit this vital cellular pathway provoke some side effects. Therefore, it is necessary to look for new therapeutic options. MicroRNAs are small molecules that modulate expression of the target genes and can be utilized as a potential therapeutic option for CRC. On the other hand, nanoformulations have been implemented in the treatment of plethora of diseases. Owing to their excessive bioavailability, limited cytotoxicity and high specificity, nanoparticles may be considered as an alternative drug delivery platform for the Hh signaling mediated CRC. This article reviews the Hh signaling and its involvement in CRC with focus on miRNAs, nanoformulations as potential diagnostic/prognostic and therapeutics for CRC.

## Introduction

Colorectal cancer (CRC) is one of the leading causes of death globally with incidence rate around two million (1). A number of factors such as the dietary habits, family history, inflammatory bowel disease, elevated body mass index, socioeconomic status, environmental and genetic factors affect the likelihood of developing CRC (2). Despite advancements in the preclinical and clinical researches, devising a suitable cure for CRC still remains bleak. Thus, researchers keep pursuing on personalized and targeted therapeutic approaches, development of efficient diagnosis/prognosis biomarkers and clinical management which could inhibit CRC development. The molecular landscape of CRC is multifarious and governed by various signaling pathways such as the hedgehog (Hh) signaling and Wnt signaling pathways which orchestrate growth and development of tumor cells (3). Hh signaling play a crucial role in regeneration of adult tissues by regulating the stem cell behavior. It also interacts with other vital signaling molecular cascades to control cellular proliferation, polarity and differentiation (4). Aberrant expression of Hh signaling is reported to be the culprit of dysregulation in cellular behavior and contribute in the onset of many human malignancies (5). Hh signaling and aberrant expression of targeted molecules promote tumor microenvironment and induce stemness of cancer cells (6, 7). Aberrant expression of Hh signaling cascade has been reported to contribute in the cancer progression and metastasis including medulloblastoma, basal cell carcinoma, breast cancer, liver cancer, pediatric soft tissue cancer, prostate, stomach, pancreas, and colon cancer (8). Invertebrates and vertebrates share common signaling molecules and mechanism in general, involving Hh ligands, patched1/2 receptor, transcriptional factors GLI-1/2/3, smoothened (SMO) as a critical signal transducer and variety of regulatory molecules (8). In mammals three Hh genes have been identified namely, sonic hedgehog (SHH), Indian hedgehog (IHH), and Desert hedgehog (DHH) which play a vital role in the embryonic development and regeneration of different organs (9). Hh signaling pathway can modulate the self-renewal of cancer stem cells (CSCs) most commonly in hematological malignancies, breast cancer, and CRC (10). There has been significant progress regarding the development of small molecule inhibitors to block Hh signaling. Several of these molecules have been included in the clinical testing stage. Yet finding a sustainable small molecule inhibitor is still a challenge. On the other hand, microRNAs (miRNAs) are small molecules that effectively regulate and modulate the expression of target genes (11). Exploring miRNAs as diagnostic tool can aid in better clinical management of CRC. Nanoformulations have been investigated in many diseases for their efficient sustainability, limited cytotoxicity, increased bioavailability and few side effects. These features have urged scientists to explore these as a therapeutic option for different cancers. In this review, we delineate Hh signaling pathway as a vital therapeutic target for CRC and shed light on the role of miRNAs that may be used as potential diagnostic marker and therapeutic target for CRC. Furthermore, the role of nanoformulations as contenders for targeted delivery of Hh signaling inhibitors for the treatment of CRC is discussed.

## Hedgehog Signaling in Cancer

**Figure 1:**
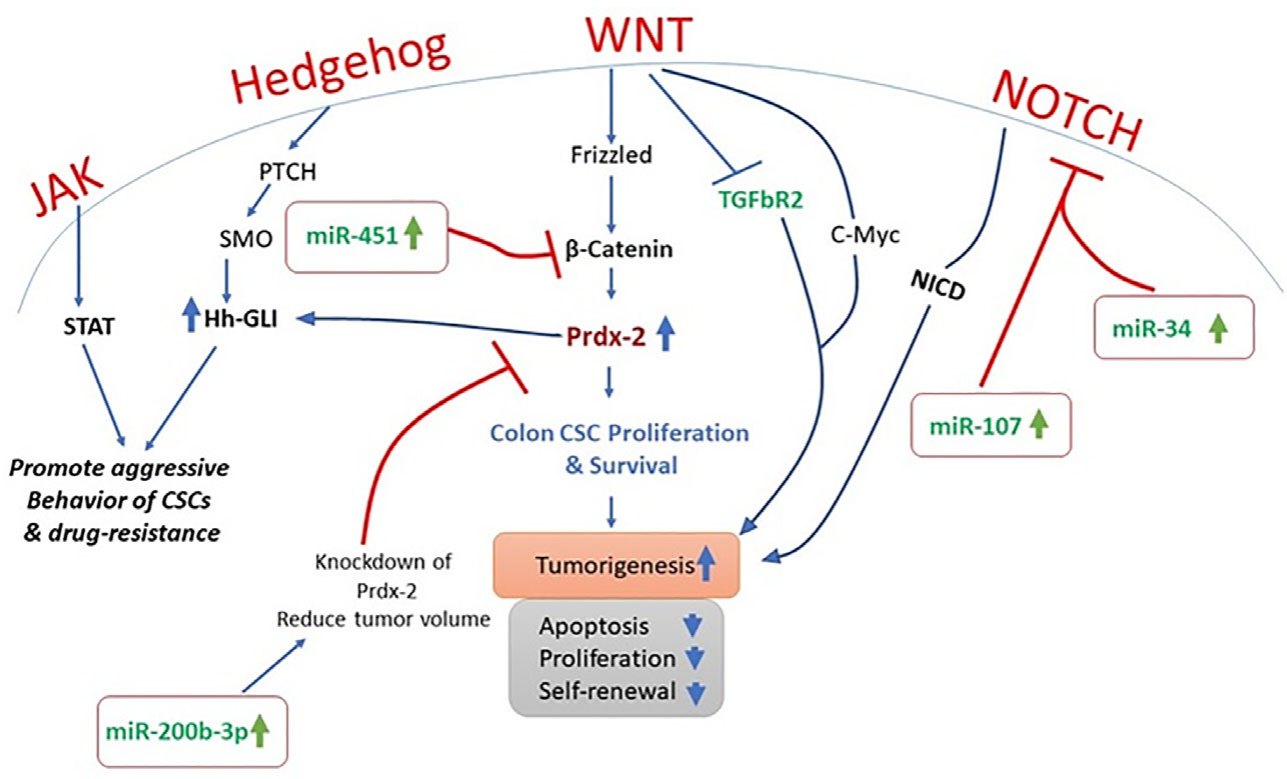
Therapeutic potential of miRNA inhibitors by targeting aberrant signaling cascades.

Molecular link of Hh signaling with cancer was reported in basal cell carcinoma when mutation in human PTCH1 gene was observed (12, 13). It was confirmed that mutation in PTCH1 is responsible for SMO activation to trigger aberrant Hh cascade activation to induce carcinogenesis (**Figure 1**) (14, 15). Similarly, increased expression of Hh targeted gene was reported in different carcinomas including meningiomas, medulloblastoma (16), small cell lung carcinoma (SSLC), gastro-intestinal cancer (17), and colon cancer. Experimental work on genetically engineered mice models exhibits that in knock-out PTCH gene mice model organism, increased expression of SMO was observed with increased tumor size. The same experimental study designed to knock out SMO in mice models revealed reduction in tumor size and metastasis (6). However due to the complex behavior of cancer onset and variation in contributing factors, no significant molecular evidence was reported in KRAS associated onset of pancreas and prostate cancer (18). Hh signaling has been associated with cellular proliferation, tissue polarity, stem cell transformation and carcinogenesis. The first molecular association of Hh signaling with cancer was established in 1996 during experimental studies on Gorlin syndrome. Hh signaling was considered as a novel therapeutic target of cancer by clinical use of Hh inhibitors (erivedge/vismodegib) and was approved in 2012 by the FDA to treat basal cell carcinoma (BCC). In this article, Hh signal in carcinogenesis and recent molecular strategies to tackle cancer cell progression using Hh inhibitors (19) were discussed.

Cancer is a complex heterogenetic disorder that transform cellular microenvironment and involves multiple and complicated crosstalk of signaling pathways. In PTCH^+/−^ mice models, inhibition of Hh signaling is an approach to limit cancer cell proliferation (20). It is also observed that STAT-3 knock-out significantly reduce the Hh-mediated delivery of BCC (21). It is widely accepted that Hh signaling has close association with the growth factor mediated pathways as Hh signaling is reported to regulate the platelet derived growth factor *ᾳ* (PDGFR-*ᾳ*). Furthermore, molecular crosstalk of Hh is reported to interplay with many other pathways including NOTCH, mTOR, Wnt, Muc5, EGF, IGF, TGF-*β*, RACK1, and PKC in different types of cancers (22–25). It has been observed that TGF-*β* regulates tumor microenvironment, while PDGFR-*ᾳ* and Notch play key role in triggering CSC (22, 25). Recent studies highlighted that inhibition of Hh signaling in cancer cells could be the iron gate for cancer therapy in many cancer types (21).

Aberrant Hh signaling is a distinguished feature of various human cancers (26). Gli1 and Gli2 are the two Hh pathway target genes that are overexpressed in the CRC (27). A gene expression microarray study conducted on 382 patients showed that Gli-1 was overexpressed in CRC patients. The expression of this target gene was responsible for the tumor recurrence and poor survival outcome in patients. In addition to this treatment of cell lines such as the HCT-116, SW480 and SW620 with SMO inhibitor GDC-0449 decreased the expression Gli target genes such as the PTCH1, HIP1, and MUC5AC. Furthermore, treatment of cell lines with GDC-0449 upregulated the expression of growth arrest gene p21 and downregulated the expression of cyclin D1 (28). The genetic silencing of SMO with 5E1, a specific antibody, prevented cell migration and invasion along with reduction in the expression of Hh target genes Gli-1 and Gli-2 (28). These findings suggest that Hh signaling affects cell plasticity, proliferation, invasion, and migration in CRC.

CSC functionality and polarity are dependent on the Hh signaling pathway (29). It has also been reported that Hh signaling induces chemotherapy and radiotherapy resistance in cancer cells (30, 31). However, Hh inhibitors are reported to promote delivery of chemo-therapeutic agent including IPI-926 (31).

Hh signaling pathway has its prime role in embryogenesis, *i.e.* cell differentiation and growth. It does not always active in all adults cells. However, in cells where stem cell development and growth is required, Hh signaling triggers on (32). Genes involved in the cellular differentiation *i.e.* proto-oncogenes and growth factors are targeted by Hh pathway, but if these pathways get activated by any mutation or if its regulation gets disturbed, then it may lead to tumor development (33). It has been observed that abnormal activation of the Hh pathway can lead to CRC. Molecular evidence realized that in CRC tissues, SHh ligand gets higher in number and increase the expression of all its downstream components, particularly SMO upregulates dramatically and difference in expression of GLI1 protein is observed. From different studies, it was revealed that SHh is a paracrine factor that works like aberrant p53 to inhibit anti-oncogenes (34). SMO activation in an abnormal way causes progression in colon cancer, and its expression was sharply upregulated in colon cancer tissues as compared to the non-cancerous colon tissues. It was observed that SMO expression is directly proportional to the stages of cancer so its level of expression can be used as an independent biomarker for liver postoperative metastasis to liver (35). Similarly, a different expression level of GLI1 was noted in normal tissue and cancerous tissue. Increased expressions of GLI1 cause activation of Hh signaling, which induce anti-apoptotic and anti-inflammatory effects on cancer cells. These alterations are potential driving forces for therapeutics to target GLI by molecular inhibitors to induce the cellular deaths of colon cancer cells. In one study it was reported that GLI1 regulation is exceptional in colon tissues and it is also related to lymph node metastasis (36). Recently, tumor suppressor gene RUNX3 is reported to play a decisive molecular role to limit endothelial proliferation in CRC. It has been observed that RUNX3 expression has inverse correlation with GLI-1 protein and it promotes GLI-1 ubiquitination in CRC. Molecular interplay of RUNX-3 gene to limit metastasis and stemness by targeting hedgehog signaling cascade could be a new contributing therapeutic agent to conquer the unbeatable fort of carcinogenesis (37). Inhibitors of hedgehog pathway are recognized in the scientific community as a therapeutic strategy for cancer treatment. Hh inhibitors hold promise for the development of a potential treatment option in CRC as its results have been proved to be very promising, suggesting that the targeting treatment of signaling pathway is a hopeful way for antitumor treatment. Therefore, the members of hedgehog signaling pathway are considered as significant therapeutic targets for the clinical treatment of colon cancer (38). The Hh signaling pathway has been seen to act as an antagonist to Wnt pathway, which is directly involved in the rapid increase of CRC cells. 90% of CRC have an active mutation in the Wnt pathway; particularly APC gene mutations are responsible agent, but Hh pathway mutations were not found as a molecular culprit in majority of CRC cells (39). There is mounting evidence that over-expression of SHh and SMO participates in the onset of multiple cancers, also recognized as SHh related carcinomas (40). Both these pathways have a significant relation between them in the occurrence and development of CRC and have numerous avenues for molecular crosstalk between the two pathways (41). The colon’s mucosa has a film of epithelial cells which gets replaced every week. It replaces large number of progenitor cells and generates plenty of new cells every day at the bottom of crypts *i.e.* small mucosal invaginations. Maintenance of the balance of cell is regulated by extrinsic signals. Morphogens, soluble proteins that make a long range of concentration gradients, produce cellular responses to target cells from a distance in a dose dependent way. It has been proved that morphogens are the main regulator in adult colon and support the notion that both Wnt and Hh pathways have significant roles in CRC (42). The metastatic transition of human colon carcinomas, which mainly occurs in the CD133^+^ epithelial tumor stem cell population, includes deregulation of the Wnt–TCF pathway and upregulation of the HH–GLI pathway (43). During this phase of metastatic transition all ligand-driven signaling pathways of Wnt are inhibited. In both CD133^+^ and CD133^−^ cells of colon carcinoma signatures of expression of gene in various stages show that activity of Wnt–TCF *i.e.* non-metastatic stops at early stages in colon carcinomas and to become metastatic, the HH–GLI works actively in stem cells. The molecular linkage was established in the deregulation and upregulation of early adenoma-like Wnt–TCF and HH–GLI1 respectively. It was experimentally proved that upregulation of HH–GLI causes downregulation of TCF and thus results in low Wnt–TCF and high HH–GLI expression in metastatic colon carcinomas. It was also observed that silencing of TCF induces the HH–GLI signaling. The high regulation of Wnt–TCF causes transcription repressor GLI3 and high regulation of HH–GLI causes repression of Wnt–TCF and GLI3 (44).

Drug resistance, tumorigenesis, tumor progression, metastasis, and tumor recurrence are the key functions that are regulated by the CSC (45). These are subpopulations of cancer cells with the ability of self-renewal. The Hh signaling pathway has been reported to be involved in the activation of CSCs in various neoplastic tumors such as the glioblastoma, leukemia, and myeloma (46). The activated stem cells have been demonstrated to play a pivotal role in the progression, metastasis, and recurrence of tumors in colon, breast, liver, and pancreatic tissues (47). In addition to its involvement in regulating the CSCs, the Hh signaling along with the SMO and Gli signaling pathways promotes cell migration, growth, and self-sustenance of CSCs (48). The non-canonical Hh-signaling has been reported as a crucial mediator for the survival of CSCs (49). Both the canonical signaling and non-canonical signaling are pivotal in regulating the expression of key genes involved in growth and proliferation of cells (45). Accumulating lines of evidence have reported the fact the aberrant non-canonical hedgehog signaling can trigger uninterrupted cellular growth in CRC. Zhang et al. demonstrated that both SMO and Gli proteins were overexpressed in colon cancer cells and colonic adenoma tissues (38). The SMO expression has been related to prognosis and tumor status in CRC patients. The CSCs are pivotal in stemness and growth of CRC. New studies have begun to shed light on the fact that non-canonical Hh signaling and Wnt signaling are the two key molecular cascades that are disrupted in CRC stem cells. Both canonical and non-canonical Hh-signaling positively and negatively regulates the expression of Wnt in CRC stem cells. Regan et al. demonstrated that non-canonical Hh signaling had a positive role in maintaining growth and differentiation of CRC stem cells. Moreover, continuous overexpression of non-canonical Hh signaling promoted resistance in CRC stem cells and increased their survival in a PTCH1-dependant, Gli-independent manner. In addition to this, SMO dysregulation has been affiliated with CSC growth and differentiation targeting; the dysregulated SMO can be a potential target for the treatment of CRC (50). A specifically designed Hh signaling antagonist GDC-0449 (Vismodegib) has been reported to suppress growth and trigger apoptosis in colon cancer cells *via* downregulating the expression of Bcl-2 (51). Another study confirmed that GDC-0449 has the ability to initiate apoptosis, decrease cellular plasticity and invasiveness of CRC (28). Altogether these findings indicate that non-canonical Hh-signaling has a regulatory role in progression and spread of CRC *via* CSCs modulation. Cancer is a multifactorial disease. There are number of factors such as the age, genetic predisposition, alteration in the genetic framework, diet and habits that can trigger tumorigenesis (52). Studies over the past decades have evidenced the involvement of various mutations in the signaling machinery that contribute towards development of cancer (53). Development of CRC like several other tumors involves mutations in the signaling machinery. Mutations in KRAS, MYB, and BRAF are the most critical mutations that trigger tumorigenesis and can be targeted for therapeutic purposes (54–57). The role of Hh signaling in CRC is still questionable. The exact mechanism by which Hh signaling triggers growth and proliferation, invasiveness and metastasis in CRC still requires aggressive research. The scientific community seems divided on the role of Hh signaling in CRC. Accumulating lines of evidence have suggested that Hh signaling has the following implications in CRC: 1) Hh signaling is expressed variably in CRC, and CRCs as different components of the Hh signaling machinery are expressed differently. 2) Hh pathway can trigger mutations in CRC. 3) Hh signaling plays a role during the transformation of the cells from adenoma-to-adenocarcinoma. 4) The SMO has the most crucial role in the regulation of carcinogenesis of CRC (58, 59). Taken together, it can be evidenced here that the Hh role in CRC still requires plenty of research.

## Role of miRNAs in Colorectal Cancers

MiRNAs are short non-coding single-stranded nucleotide sequences (60), which affect almost all physiological processes in cells such as development (61, 62), proliferation (63), differentiation (64), apoptosis (65), signal transduction (66) and many more. The altered expression patterns of miRNAs are tightly linked with a wide range of anomalies including various cancers; thus miRNAs screening could serve a very good therapeutic and diagnostic tool in molecular biology (67). Till date, more than 25,000 miRNA sequences have been identified, and this number is growing fast amid current research interests in miRNAs all over the world. According to an estimation, 3–4% of human genome comprises of miRNAs (68). These miRNAs interfere with numerous key regulators of cellular processes by binding with post-transcriptional products. For this reason, miRNAs are considered as important biomarkers for many cancers including CRC (42). In this section, we shall focus on miRNAs which interact with Hh signaling and may affect CRC. There is a long list of miRNAs which affect CRC progression. More than 500 miRNAs have been found to be linked with CRC. Among these miRNAs few miRNAs such as miR-21 (69), miR-143, and miR-145 are reported most frequently and are summarized in (70). These miRNAs interact through various signaling pathways. For example miR-143 significantly inhibits KRAS which ultimately suppresses CRC (71). However, another study has shown the opposite phenomenon where reduced levels of miR-143 expression were detected in CRC tissues. Interestingly transfection of cells with transient miR-143 turns the cells to mimic SW480 cells, a CRC cell line, resulting in increased levels of cell proliferation and apoptosis (72). Thus, we may say that the role of particular miRNAs may also vary depending upon the cell type. On the other hand, the role of miR-145 remains much consistent as CRC suppressor in many studies. There has been a reverse interaction between erythroblast transformation-specific (ETS)-related gene (ERG) and miR-145 in CRC. Increased ERG results in decreased miR-145 levels and promotes CRC. The overexpression of miR-145 suppresses CRC by decreasing expression of ERG (73). A similar relation between P21-activated kinases 4 (PAK4), and miR-145 was also observed where miR-145 appeared to downregulate phosphorylation level of LIMK1 and cofilin in SW1116 cells through PAK4 (74). miR-224 activates the Wnt/*β*-catenin signaling by deregulation of GSK3*β* and SFRP2 to translocate *β*-catenin in CRC (75). Similarly, miR-361 is also downregulated in CSC (65). miR-150 is negatively correlated with circular RNA named Circ-ZNF609 and important transcription factor of hedgehog signaling *i.e*. Gli1 in HCT-116 cells (76). Another study stated that overexpression of miR-150 positively affect the EMT and subsequent downregulation of Gli1, further confirming the role of miR-150 in CRC through hedgehog signaling (77). Similarly, miR-142-3p appeared to promote cell invasion in CRC by upregulation of RAC1 (78). There are miRNAs also targeting other key regulators of hedgehog signaling. One such miRNA-378 inhibits SUFU and promotes cell survival and tumor growth (79). Another molecule, miR-146a, activates the Wnt pathway and stabilizes *β*-catenin, thereby promoting CRC by regulating the symmetrical cell division by a feedback loop of Snail-miRNA-146a-*β*-catenin (80). All variants of hedgehog pathways work upstream of epithelial-mesenchymal transition (EMT) (81). A number of miRNAs regulate EMT in CRC such as miR-29c which has been shown to be remarkably downregulated and also associated with metastasis and significantly shorter patient survival and this effect was reverted by transient expression of miR-29c (82).

MiRNAs are crucial molecular factors to regulate post-transcriptional processing, and more than 60% protein coding genes are expected to regulate miRNAs, and their dysregulation is often reported to trigger different human disorders including cancer. In recent years, many reports highlighted the significance of miR-34a as tumor suppressive molecular entity. It has been figured out that miR-34a has inverse relation with the cancer progression, and the expression of miR-34a declines with the increased progression of cancer and *vice versa* (83).

*Scutellaria barbata* (SB) is a natural compound and has been used for years as a potential compound among traditional Chinese medicines against multiple cancers. *In vitro* and *in vivo* clinical trials have proved that its ethanol extract of SB is an effective agent to induce apoptosis and limit cancer cell proliferation (84). Ethanol extract of SB has been found effective against human CRC HCT-8 cells and regulates miR-34a expression. Molecular assay confirmed that SB extract upregulates the miR-34a expression and negatively regulates the Bcl-2, Notch and Jagged-1 gene expression. miR-34a mediated down-stream targeted gene regulation plays a decisive role in apoptosis and limits cancer cell proliferation. In one of the studies, exogenous inactivation of miR-34a by using anti-miR-34a oligonucleotide triggers Bcl-2, Notch1/2, and Jagged-1 genes and promotes cancer growth (84, 85). Activation of miR-34a has been associated with regulation of various cellular processes including apoptosis, proliferation, and invasion (85). Molecular evidence also established a link of miR-34a with downregulation of Notch1/2 in colorectal CRCs (86).

miR-449a has been documented as tumor suppressor gene and has been closely associated with SATB2 in different cancer types including CRC cells. SATB2 could be used as diagnostic marker for CRC and has comparative negative association with miR-449a. It has been noted that in CRC xenograft mouse models, increased expression of miR-449a promotes apoptosis by negatively regulating the expression of SATB2 (87). Similarly, molecular link has been established to understand the transcriptional deregulation of SMO by miR-326, and it was observed that upregulation of miR-326 negatively regulates SMO protein to induce apoptosis and limit cellular proliferation (88, 89).

Aberrant expression of GLI-1 (Glioma associated oncogene homolog 1) is a key culprit in the metastasis, invasion, and proliferation of various cancer cells. Ample lines of evidence have shown that expression of miR-150 declines with the pathogenesis of CRC. NCM-460 and SW-620 CRC cell lines were examined by using dual luciferase assay to decipher the molecular relation of miR-150 with GLI-1, and it has been noted that miR-150 inhibits the expression of GLI-1 protein in Hh signaling (77). Molecular evidence has proved that Hh is a cellular event responsible for structural development, cellular regeneration, and stemness. In multiple myeloma cancer (MMC), inverse relation of miR-324-5p and hedgehog signaling has been observed. Increased expression of miR-324-5p has significant inhibitory effect on SMO and GLi-1 and limits cancer stemness of cells (90). Pro-oncogenic effect of miR-212 is identified, and molecular relation was established that miR-212 induces pancreatic ductal adenocarcinoma (PDAC) by targeting PTCH-1 (91). miR-361-3P has been reported to have profound impact on different cancers including prostate cancer, breast cancer, lung cancer, and cervical cancer. To decipher the molecular interplay of miR-361-3p in retinoblastoma (RB) tissue and RB cell lines, Weri-Rb-1, and Y79, real-time PCR analysis was performed, and it is concluded that miR-361-3p expression is downregulated with cancer progression. Forced expression of miR-361-3p is reported to limit cancer cell proliferation by targeting GLI-1/3 and sonic hedgehog signaling (92). Multiple miRNA expressions have been associated with the onset of breast cancer including miR-454-3p, miR-130b-3p, miR-421, and miR-301a-3p. These miRNAs are noted to target PIEZO-2 gene. Downregulation of PIEZO-2 gene in breast cancer has been molecularly linked with estrogen and progesterone receptors which are responsible agents for Hh signaling cascade in breast cancer (93).

**Figure 2:**
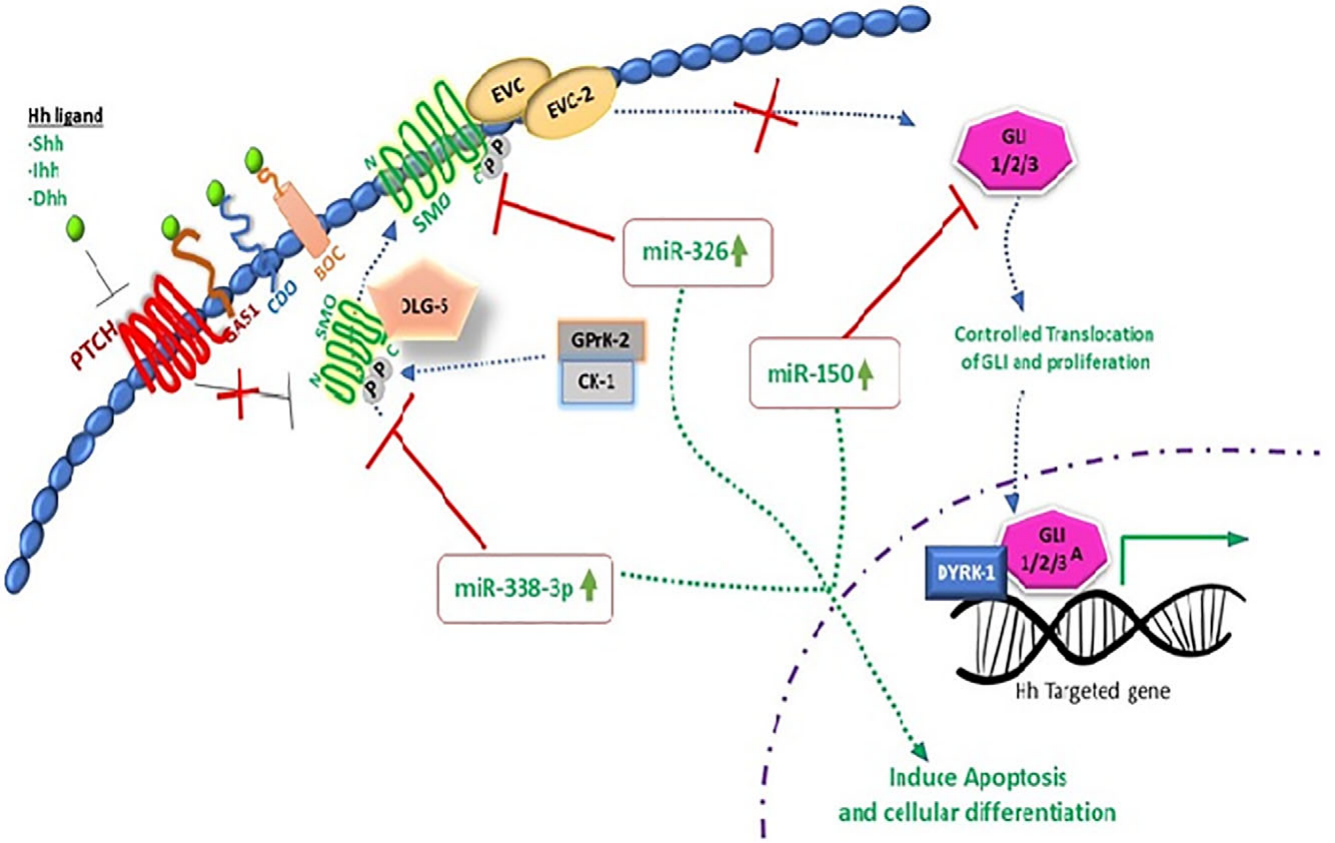
miRNAs targeting SMO oncogenic hedgehog pathway to induce apoptosis in CRC.

**Figure 3:**
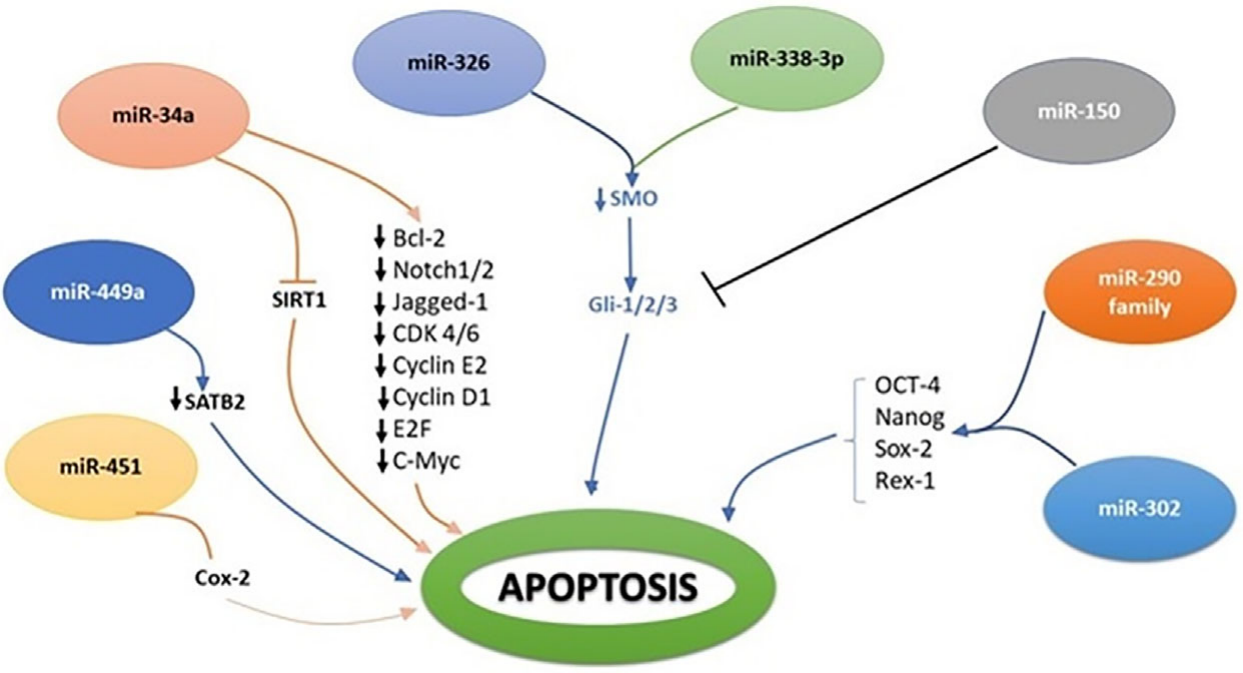
Molecular interplay between several miRNAs and their downstream target genes to induce apoptosis.

Extensive research work on miRNAs has been done in recent years to unfold the molecular complexity of carcinoma and to bridge the gap towards new and effective therapeutic approaches. miR-338-3p interaction with hedgehog pathway by using recombinant lentiviral vectors PLV-THM-miR-338-3p and PLV-THM-miR-338-3p inhibitor has been reported and successful transfection in SW-620 CRC cells was achieved. Increased expression of miR-338-3p was observed, it significantly suppresses SMO protein and inhibits proliferation ability. Molecular interplay of miR-338-3p is also confirmed by using PLV-THM-miR-338-3p inhibitor, and it was concluded that it upregulates the SMO protein expression to initiate hedgehog signaling pathway and induces CRC onset. miR-338-3p could be a therapeutic agent to suppress CRC growth by targeting SMO, (**Figures 2** and **3**) (94). Now, withstanding the fact that SMO is a possible target of miR-338-3p. 40 CRC tissue samples and 2 CRC cell lines, SW620 and SW480 were investigated to understand the corresponding protein expression of SMO and miR-338-3p by using semi-quantitative RT-PCR, western and northern blotting assays. It was established that miR-338-3p plays a significant role in metastasis and progression of CRC carcinoma (95).

Accumulation of genetic and epigenetic errors can trigger the aberrant signaling cascades. miRNAs are the critical key players to fine-tune genetic expression upon exogenous factors including DNA hypermethylation, hypomethylation, histone modification, and deacetylation. Thus epigenetic-miRNA regulatory molecular cascades are the contributing agents for onset of different types cancers (96). Tumorigenic activation of SMO by over-expression of Shh ligand is reported as responsible agent in 40% cases of human hepatocarcinogenesis (97). Molecular balance between cellular proliferation, differentiation, and renewal is modulated by epigenetic regulatory network and miRNAs. miRNAs are the functional short RNAs that control stemness of cancer cells and promote stem cell self-renewal (97). CSCs are believed to be the critical source for tumor initiation. It has been reported that SHH signaling has reprograming potential for epigenetic memory power within CSCs to modulate cancer hallmarks. miR-302-367 clusters regulate cellular plasticity molecular cascade by engaging cyclin-D1, CDK-4, OCT-4 and SOX-2 genes (98).

## MicroRNAs as Master Regulator of Stemness and Metastasis

Cancer stem cells are the cells with self-renewal potential within the tumor and are the key responsible agent for radio- and chemo- resistance behavior of cancer cells. Increasing evidence strongly suggests that CSCs are the responsible factor for the onset of carcinogenesis in many human cancer types as cancer cells have self-renewable stem cell like characteristics. Several microRNAs expression have been associated to regulate cancer stemness pathways and its downstream targeting genes (115). Tumor cells were believed to derived from normal stem cells or progenitor cells that undergo genetic or epigenetic modification and transform themselves into CSCs by attaining unlimited self-renewable and differentiation potential (116, 117). Recent findings provide striking evidence that dysregulation of miRNAs regulates CSC characteristics and induces tumorigenesis, and multi drug resistance behavior of cancer cells. The four basic stemness transcriptional factors, OCT4, Nanog, Sox2, and Rex1 are responsible entities for cellular pluripotency and differentiation (118, 119). Recent molecular evidence supports the notion that members of the miR-290 family provide protective defense again differentiation defects in ESCs and play a key role for OCT-4 stability (120). miR-302 family is reported to limit the self-renewal ability and cellular differentiation by regulating the expression of key genes in stem cells. miR-34 family members contribute effectively in P53 dependent reprograming of human ESCs, and it has been noted that loss of functional ability of miR-34 is associated with the upregulation of pluripotency genes including N-Myc, SOX2, and NANOG (115). miR-34a is also reported to modulate neural differentiation by targeting SIRT1. It has been widely accepted that miRNAs are the potential contributors to regulate stem cell properties and stemness of cancer cells (115).

To understand the molecular underpinning of cancer stemness, CSCs were isolated from SW-1116 colon cancer cell lines with both CD133^+^/CD44^+^ and CD133^−^/CD44^−^ surface phenotype antigen for comparative analysis. Researcher found 62 differentially expressed miRNAs in cancerous and non-cancerous stem cells and noted 31 miRNAs overexpressed including miR-29a and miR-29b, as well as 31 miRNAs under-expressed including miR-449a, miR-4524, and miR-451 (117). Exogenous expression of miR-451 declines the self-renewable capacity of stem cells and decreases multi drug resistant potential of cancer cells. Induced expression of miR-451 negatively regulates COX-2 gene that plays a decisive role in Wnt cascade activation and is believed to act as complementary factor for CSC activation. Wnt pathway has a key association with intestinal stem cell regulation and reported to linked with colon cancer onset (117, 121). Inhibition of Wnt pathway leads to the degradation of *β*-catenin in the cytoplasm and is unable to initiate epithelial renewal. Increased expression of miR-21 is noted to induce stemness by regulating Wnt activity and thus initiates carcinogenesis by inhibiting the tumor suppressor gene, TGF-βR2, that is a key regulatory gene for cellular differentiation (122). One of the fundamentals signaling pathway to regulate colon stemness is Notch. Notch cascade activation is believed to induce cellular proliferation of progenitor cells. miR-34a is shown to downregulate Notch signaling activity and regulate cellular differentiation of targeted cells and colon stem cells (123) (**Table 1**).

**Table 1:** MicroRNA mediated control of CRC stemness and progression.

MicroRNA	Expression pattern	Target	Function	Reference
miR-150	Upregulated	Gli-1, 2 & 3	Downregulation of the expression of Gli-1/2/3 in CRC thus prevents apoptosis	(77)
miR-34a	Upregulated	Bcl-2, Notch1/2, Jagged 1,2 CDK, Cyclin-D, E2F, c-Myc and Cyclin-E2 SIRT1	Downregulates the expression of Bcl-2, Notch1 and 2, Jagged 1,2, and CDK, Cyclin-D, E2F trigger apoptosis in CRC	(124)
miR-338-p	Upregulated	SMO, DLG5, GPrK-2 and CK1	Downregulates/inhibits the expression of SMO, DLG5, GPrK-2, and CK1 trigger apoptosis CRC	(94)
miR-150	Upregulated	Gli-1/2/3	Downregulates/inhibits the expression of Gli-1/2/3 thus preventing proliferation in CRC	(77)
miR-326	Upregulated	SMO, EVC-1/EVC-2	Downregulates/inhibits the expression of SMO and EVC-1/EVC-2 in CRC	(88)
miR-290/miR-302	Upregulated	OCT-4, Nanog, Sox-2, and REX-1	Upregulates the expression of OCT-4, Nanog, Sox-2, and REX-1 and triggers apoptosis in CRC	(125)
miR-21	Upregulated	PTEN, AkT	Increases stemness and invasiveness in CRC *via* upregulation of Akt pathway	(69)
miR-148a	Downregulated	Wnt/*β*-catenin	Reduces cancer stemness in CRC cell lines	(126)
miR-137	Downregulated	Doublecortin-like kinase 1 (DCLK1)	Downregulates the DCLK1 and suppresses tumor growth in CRC	(127)
miR-372/373	Upregulated	Nanog/SMO	Downregulates MAPK/ERK and VDR signaling thus increases cancer stemness in CRC	(128)
miR-196b-5p	Upregulated	STAT3	Upregulates the expression of Nanog, SOX2 and OCT4 increases the stemness profile of CRC stem cells and triggers drug resistance.	(129)
miR-195-5p	Downregulated	SOX2, CD133	Suppresses the stemness and chemo-resistance in CRC CSCs *via* modulation of key signaling pathways proteins such as Notch2 and RBPJ.	(130)
miR-199a/b	Downregulated	Glycogen synthase kinase 3 β (GSK3 β)	Increases chemo-resistance in CRC *via* modulation of Wnt/*β*-catenin and ABCG2 signaling pathway	(131)
miR-31	Upregulated	EphB2/EphA2 signaling	Increases cancer stemness *via* modulation of the EphB2/EphA2 signaling in CRC CSc.	(132)

### Putative Markers for CRC

LGR5 is a member of G-protein coupled receptor that can interact and trigger activation of Wnt signaling *via* binding to furin-like repeat FU2 domain of R-spondin (133). LGR5 has been reported to be a putative marker for CRC stem cells. It has come to light less lately that LGR5 triggers the activation of both Wnt and TGF-*β* signaling in cancer stem cells. Overexpression of LGR5 increases drug resistance and cancer stemness in both brain tumors and CRC. LGR5 has been reported to have high expression in most of the CRC cell lines and adenomas but this overexpression has nothing to do with progression of tumor as presence of LGR5 increases cell-cell adhesion which in turn promotes stemness and hampers invasiveness and migration (134). Experimentation conducted on the triple positive cells having LGR5-positive subpopulations demonstrated peculiar characteristics of self-renewal, differentiation, colony formation, tumorigenicity, and stemness (135). These findings suggest that LGR5 is a putative marker of CRC stem cells. A transgenic mice experiment confirmed the status of LGR5 as CRC stem cell marker. Addition of suicide gene to a transgenic mice genome that was activated in the presence of overexpressed LGR5 and tamoxifen resulted in the death of LGR5 rich colorectal stem cells. The absence of tamoxifen resulted in differentiation of LGR5 CSCs (136). From these findings it can be concluded that LGR5 is a putative CSC marker that should be considered as potential target for advanced grade CRC and such CSCs must be eradicated before the start of any combinational therapies for CRC. CD44 is a surface protein responsible for interaction between cells and also plays vital role in the adhesion and migration of the cells (137). The CD44 has a specific binding site for hyaluronic acid which facilitates interaction with selectin, osteopontin, fibronectin, laminin, and collagen in the extracellular matrix (138). The binding of hyaluronic acid with CD44 results in the activation of epidermal growth factor receptor family kinases such as the MAPK and PI3/AkT that in turn promotes growth and proliferation in various cancers (138). Majority of CSCs population have CD44 surface markers along with other cell surface markers that increase invasiveness and stemness (139). Considering its abundance in CRC tumorous stem cells it has been reported as putative marker for the detection of invasiveness and metastasis. Cluster differentiation 24 is an emerging biomarker for CRC (140). Overexpression of CD24 is affiliated with tumor differentiation, invasion metastasis (141). In addition, CD24 overexpression also promotes poor survival rates in the patients with CRC. These findings suggest that CD24 involvement increases stemness in CRC stem cells and may be used as a prognostic marker for patients with CRC.

## Nanotherapeutics as an Approach for the Treatment of CRC

Hh pathway can be targeted by the specific inhibitors at various sites. Therefore, inhibitors of Hh pathway are useful anti-cancer agents. Till now, several small molecules inhibitors have been developed tested for their inhibitory effects on Hh signaling pathway. A natural alkaloid cyclopamine obtained from the corn lily V. californicum is the first Hh inhibitor reported. Cyclopamine impedes the functioning of smoothened *via* inhibiting its attachment to the heptahelical bundle (142). However, cyclopamine has several drawbacks such as the limited bioavailability, chemical instability, and shorter half-life. Therefore, it cannot be considered as potential therapeutic target. Several small molecule antagonists such as the SANT1, SANT2, SANT3, SANT4, CUR-61414, and GDC-0449 have been synthesized and evaluated in pre-clinical models for their anti-cancer activity in various solid tumors (143). The Hh small molecule inhibitors were first evaluated in basal cell carcinomas. Vismodegib a small molecule inhibitor of SMO is the first reported drug used for the treatment of basal cell carcinoma (144). In comparison to the cyclopamine, vismedegib was efficient to culminate cancer growth in both advanced and metastatic basal cell carcinomas. Over the years new therapeutic interventions in the development of SMO for Hh signaling antagonists such as LDE225 also known as the sonidegib has increased the drug efficacy *via* increasing tissue absorption and better penetration in the blood brain barrier for skin cancer and brain tumor respectively (144). There are several SMO antagonists designed to inhibit Hh pathway are in clinical trials that specifically target medulloblastoma, ovarian cancer, pancreatic cancer, and colon cancer (145). Yet the clinical success of these antagonists is still limited. In order to understand the effects of these SMOs on hedgehog dependent inhibition of CRC further investigation is required for finding suitable and effective drug. In recent years huge development in the field of nanotechnology has enabled us to devise efficient therapeutics for various diseases (146). In addition to this, nano-carriers have greater efficiency in delivering drug to target site with limited cytotoxicity. These observations have urged scientists to seek more efficient nano-drug delivery systems that can hamper cancer progression and increase apoptosis. There have been some serious drawbacks of utilizing SMO as inhibitors of Hh signaling (147). The SMO antagonists have poor bioavailability, drug resistance and non-specific activation of Gli (148). Nanoformulations can address these drawbacks by increasing bioavailability, reducing drug resistance and specific activation of Gli. Based on current data there are two types of nano-based Hh signaling inhibitors: Natural Inhibitors and synthetic inhibitors. Cyclopamine comes under the list of natural inhibitor that has faced severe criticism because of its limited bioavailability, poor solubility, and several side effects. However, nanoformulations of cyclopamine have reduced these obstacles. It has been reported that cycolopamine loaded lipid nanoparticles (NPs) efficiently reduced the growth of radiation therapy treated breast and pancreatic cells (149, 150). In another study, polymeric nanoparticles designed to carry both cyclopamine and doxorubicin reduced the growth in orthotropic breast cancer model (151). The polymeric nanoformulations of cyclopamine and paclitaxel successfully cured prostate cancer (152) and pancreatic cancer (153) in combination with chemotherapy in mice. A biomimetic nanoparticle delivery system having cyclopamine encapsulated in erythrocyte membrane camouflaged PLGA resulted in super enhanced bioavailability of cyclopamine. Moreover, a combination of biomimetic NPs with paclitaxel NPs increased the delivery of paclitaxel to the tumor tissue increased tumor profusion and inhibited tumor growth *in vivo* (154). Vismodegib an FDA approved natural inhibitor for Hh signaling pathway has limited solubility and bioavailability. The polymeric nanoformulations for vismodegib have resolved these issues. The encapsulation of vismodegib in SN38 pro-drug polymer which is an active metabolite of irinotecan to treat pancreatic ductal carcinoma resulted in decreased tumor growth and reduced fibrosis. In addition to this SN38 NPs facilitate the inhibition of Hh signaling which is crucial for the communication between tumor and stromal cells. SN38 NPs provided better diffusion for vismodegib thus prevented the drug resistance. SN38 NP encapsulation of vismodgib suppressed Gil1 expression in the tumor microenvironment of xenograft model suggesting the fact that SN38 NPs could aid in restoring normal drug resistance of the tumor cells (155). In another study, pH-responsive polymeric NPs containing vismodegib and gemcitabine inhibited growth of pancreatic cancer cells (156). From these findings, NP mediated drug delivery of Hh signaling inhibitors can be used a potential tactic to trigger chemotherapy. Apart from cyclopamine and vismodegib, several other natural Hh signaling inhibitors have been reported to be delivered by the nanofomulations. Anthothecol carrying PLGA nanoparticles have been reported to suppress proliferation and colony formation of pancreatic cancer stem cells through modulating the activity of the Gli-DNA binding (68). Another study confirmed that *α*-mangostin carrying PLGA nanoformulation disrupted the Gli-DNA binding activity in pancreatic cancer cells. This resulted in decreased growth, development, and metastasis of pancreatic cancer stem cells (157). Nanoformulation of glabrescione B has been reported to show tremendous anti-cancer activity in a Hh dependent manner (158). Nano-carriers have been employed in the delivery of the synthetic inhibitors of Hh signaling pathway. Quinacrine a synthetic inhibitor of Hh signaling when loaded into NP formulation triggered the recruitment of GSK-3*β* and PTEN which induced the apoptosis in cancer stem cells. In addition to this qunacrine loaded NPs also reduced the expression of Gli vital for the self-renewal of CSCs (159, 160). PLGA NPs encapsulating the HPI-1 a specific inhibitor of Gli1 prevented growth and metastasis of hepatocellular carcinoma mice model. Moreover, HPl-1 delivery reduced the expression of CD133^+^ cells a type of CSCs in hepatocellular carcinoma (161). A combination of the NPs and gemcitabine reduced cellular growth in xenograft model of pancreatic cancer in a ligand dependent paracrine activation of Hh signaling pathway (162). GANT61 a specific Gli1 inhibitor when encapsulated in PLGA NPs prevented the translocation of Gli-1 to the nucleus and reduced the growth of CSCs (163). Although there has been slight progress towards the utilization of nano-carriers as a module to treat Hh mediated CRC, these observations are in favor that nanoformulations could be used as small molecules Hh inhibitors to cancer. It has come to light less lately that nanoformulations can be used as a carrier for the targeted delivery of the miRNAs. There are different types of nanoparticle based formulations that are being used for this purpose. Lipid nanoparticles, extracellular vesicles, minicells (genetically developed from bacteria), dendrimers, polyamidoamine (PAMAM), and inorganic materials such as the silica, gold, and silver nanoparticles have been extensively synthesized for delivering specific miRNAs to targeted tumors. Oshima et al. have developed an *in-vivo* delivery system to target liver and CRC. They specifically designed nano-scale coordination polymers for the effective delivery of oligometastatic miR-655-3p. Their findings revealed that co-delivery of miR-655-3p along with oxaliplatin reduced tumor growth (164). Yang et al. successfully formulated polyethyleneimine/hyalouronic acid mesoporous silica nanoparticle loaded with oxaliplatin and miR-204-5p. This nanoformulation enhanced the apoptosis and therapeutic efficacy in HT-29 cell lines (165). Altogether these findings suggest that nanoformulations are a suitable platform for the delivery of miRNAs and can increase the therapeutic efficacy in CRC (**Table 2**).

**Table 2:** CRC inhibitors and nanoformulations.

Nanoformulation	Ligand	Target	Cell line(s)	Reference
Nanosized maghemite particle	Antibody	CEA	HCT-116	(99)
Dextran- and PEG-coated superparamagnetic iron oxide nanoparticles (abf-SPION)	scFv	CEA	LS174T	(100)
Conatumumab (AMG 655)-coated nanoparticles	Antibody	DR5	HCT-116	(101)
Photosensitizer meso-Tetra(N-methyl-4-pyridyl) porphine tetra tosylate chitosan/alginate nanoparticles	Antibody	DR5	HCT-116	(102)
Gold and iron oxide HNPs	scFv	A33 antigen	SW1222 & HT-29	(103)
Poly(lactide- coglycolide) nanoparticle loaded with camptothecin	Antibody	Fas receptor (CD95/Apo-1)	HCT-116	(104)
Chitosan nanoparticles loaded with 5-ALA	Folic acid	FR	HT29 and Caco-2 colorectal cancer cell lines overexpressing folate receptor	(105)
FA-CS conjugates nanoparticles	Folic acid	FR	HT-29	(106)
HPMA-copolymer-doxorubicin conjugates	Peptide GE11	EGFR	HT-29, SW480 and A431	(107)
T22-empowered protein-only nanoparticles	18-mer peptide T22 (T22-GFP-H6)	CXCR4	HeLa	(108)
Chitosan nanoparticles encapsulating oxaliplatin (L-OHP)	HA	HA receptor	HT-29 in C57BL mice	(109)
MSN	Coated with poly-(L-lysine) and HA	CD44 receptor	HCT-116	(110)
rHDL nanoparticles loaded with siRNA	Apo A-I	SR-B1	Model colorectal cancer metastasis in mice (HCT-116)	(111)
HA-lipid shell nanoparticles	Gene therapy, core shell	P21-saRNA-322	HT-29	(112)
Survivin siRNA	Cationic LCLs, Gene therapy	Lipolex	LoVo	(113)
Exosomes	Gene therapy	miR-128-3p	HCT-116oxR	(114)

Apo A-1, Apolipoprotien A-1; CEA, Carcinoembryonic antigen; CXCR4, CXC chemokine receptor 4; DR5, Death receptor 5; FR, Folate receptor; HA, Hyaluronic acid; HNP, Hybrid nanoparticle; EGFR, Epidermal growth factor receptor; DR5, Death receptor 5; HA, Hyaluronic acid; Scavenger receptor type B1.

### Oral Nanotherapeutics

**Figure 4:**
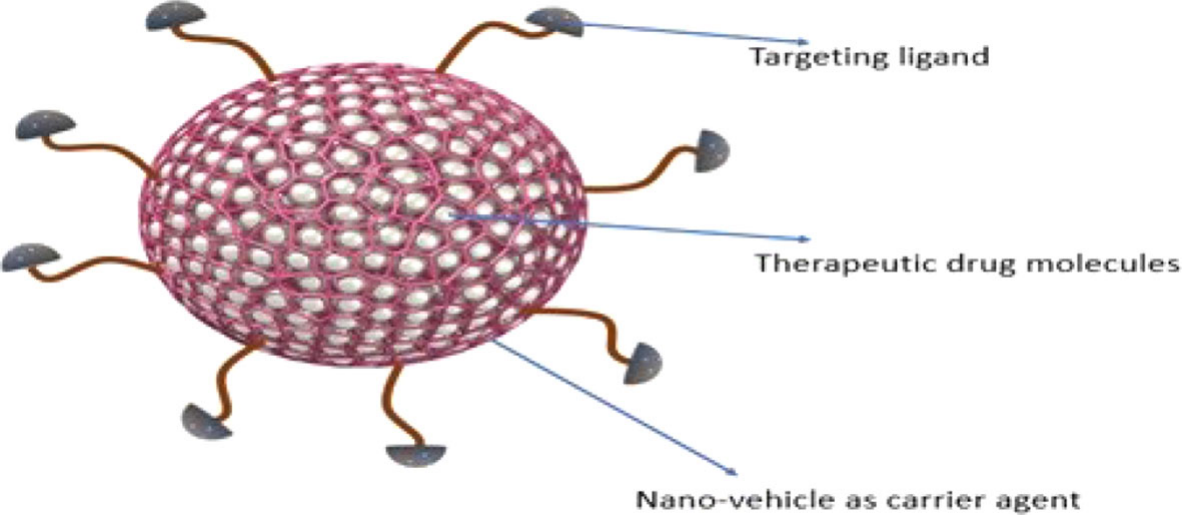
Schematic illustration of nanotherapeutic components.

Nanotherapeutics is one of the promising strategies that offer dynamic surface functionalized modifications to improve targeted drug delivery and to limit the adverse effects. Nano-platform for drug designing offers potent routes to enhance drug profile. Nanotherapeutics are generally comprise of three core elements including nano-vehicle as carrier agent, target ligand and therapeutic drug molecule (**Figures 3** and **4**) (166). Different nanotherapeutics including lipid nanoparticles, organic and inorganic nanoparticles, polymeric nanoparticles, nanocrystals and plant derived nanomaterials have been used clinically including Taxel^®^, Lipo-Dox^®^, Abraxene^®^, Abraxene^®^, Marqib^®^, Onivyde^®^ *etc.*, and there is large number of nanotherapeutics in pre-clinical and clinical trials that can be fabricated to target Hh signaling cascade to reduce cancer cell proliferation (166, 167).

**Figure 5:**
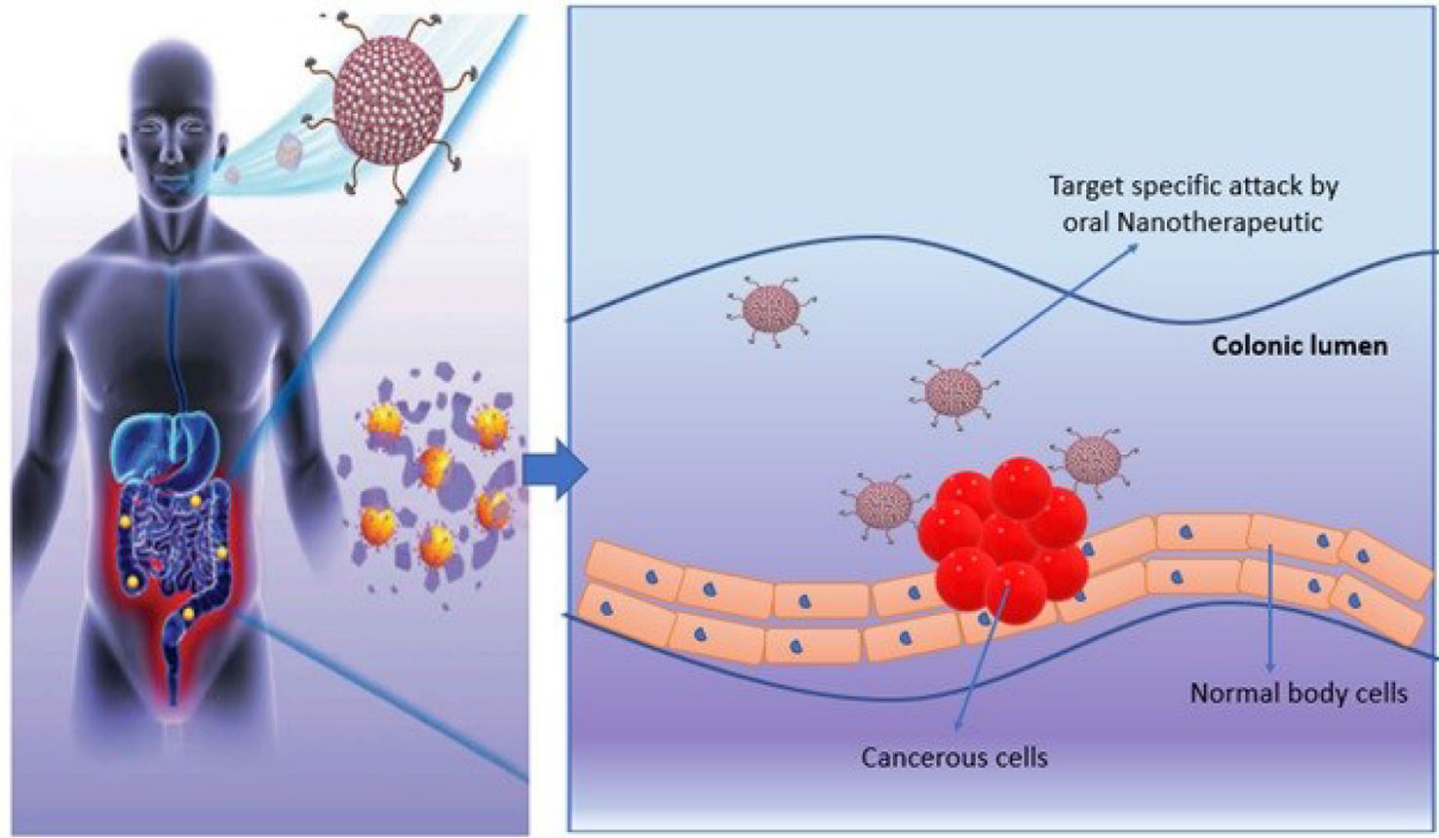
Schematical representation the passage of oral nanotherapeutics to treat colon cancer.

Oral chemotherapeutics are reported to have multiple limitations that demand novel alternative therapeutics for cancer treatment. Recently, the concept of oral nanotherapeutics paved the avenue in pharmaceutics towards more stable and high tumor targeted therapy with minimize adverse effects. A successful pre-clinical attempt has been performed by synthesizing redox nanoparticles (RNPs) for colon cancer treatment. This novel RNP contains nitroxide radicals for antioxidant activity and to scavenge ROS (reactive oxidative species) in cellular microenvironment. RNPs are specialized to accumulate in colonic mucosa and targeted cancer cells predominantly. These specialized oral-nanotherapeutics are also reported to limit cellular toxicity issues upon prolong exposure and is significant agent to inhibit tumor growth (**Figure 5**). It has been noticed that synergistic effect of RNP and conventional therapeutics can suppress adverse effects in gastro-intestinal tract and is an ideal candidate for future with significant potential in the existing pool for cancer treatment (168).

Various attempts have been made for successful oral delivery of targeted nanotherapeutics to treat cancer including bowel inflammatory cancer and colon cancers. Engineered chitosan based amphiphilic muco-adhesive drug-delivery strategies have been examined in *in-vivo* therapeutic studies. In one of the recent studies, SN38 (7-ethyl-10-hydroxycamptothecin) nanoparticles and water insoluble curcumin are proved to be a significant candidate to limit carcinogenesis and shrink tumor diameter >4 mm. Thus, bio-adhesive chitosan based stable colloidal nanotherapeutics is a novel and reliable approach to improve the outcome of colon cancer treatment (169).

There are a number of inhibitors related to Hh signaling namely sonidegib, saridegib, itraconazole, BMS-833923, LEQ-506, Taladegib, Glasdegib, TAK-441, Vismodegib, and several others (**Table 3**). But a very limited data is available regarding the use of these inhibitors for CRC. In addition to this majority of work done so far is on Wnt/*β*-catenin and mutations in this pathway. Therefore a lot of potential works need to be done against the implementation of such inhibitors in the clinical trials (**Table 3**).

**Table 3:** Representation Hh signaling inhibitor under clinical trials.

Therapeutic Agent	Development Phase	Type of Cancer	Trial Identity
**Vismodegib**	Phase I	Myelomas	NCT01330173
	Phase II	Medulloblastoma	NCT01878617
	Phase IV	Basal cell Carcinoma	NCT02436408
	Phase II	Colorectal Cancer	NCT00636610

## Future Perspective

The Hh pathway is activated during regeneration and tissue repair in adults. Compelling pieces of evidence have indicated that inhibition of Hh pathway can prevent tumor progression and increased apoptosis. However, the clinical outcomes of such inhibition are unsatisfactory. The tumor heterogeneity and complex signaling crosstalk are the two major stumbling blocks that challenge the specificity of a specific Hh inhibitor. A tumor cell can trigger multiple signaling pathways simultaneously that can hamper the anti-proliferative abilities of a single inhibitor. Therefore, an outlook for new inhibitors of Hh signaling pathway that can hamper the activity of interconnected pathways is necessary. miRNAs have been reported to regulate the expression of vital genes involved in the proliferation and spread of CRC. They have been extensively examined for their putative role as diagnosis and prognosis markers for stratification of risk groups. Exploring the interplay between miRNAs and Hh signaling can aid in the development of therapeutics for Hh mediated CRCs. In addition to this miRNAs can regulate the cancer stem cells proliferation and metastasis (170). Therefore, they can be utilized as a probe to investigate cancer stemness and drug resistance in CRC stem cells. miRNAs modulating the expression of the proliferative genes is a hallmark in CRCs. A suitable drug delivery system can transfer miRNA modulating moieties to the target cell can impede the proliferative capabilities. Development of such delivery system will revolutionize therapeutics. Considering such scenario, nanoformulations can be a suitable platform for the treatment of various malignancies including cancer. Nanoformulations are advantageous because of their specificity, low toxicity, limited side effects, and enhanced bioavailability of the cargo such as various natural compounds *i.e.*, berberine, paclitaxel curcumin, and SMOs. Hh signaling can be targeted with nanoparticles, but there are several drawbacks affiliated. Drug resistance is the major hurdle with the devising of NPs for Hh signaling. The complex interaction of Hh pathway makes it difficult to be targeted with nanoformulations of SMO. It has been reported that SMO nanoparticles were unable to hamper tumor growth when Gli was activated *via* non-canonical Hh signaling. In addition to this the interaction of Hh signaling with other molecular cascades such as the Wnt and Notch also affect the targeting capacity of small molecular inhibitors of Hh signaling. The nanoformulations have to overcome cellular resistance which is an effective barrier for suitable nano-drug delivery which can be overcome by the combination of nanoformulations such as pegylated liposomes and formulations that prevent quick release of cargo (Chitosan). Compelling research has dictated the fact that only 0.7% of the total nanoformulation reaches the solid tumor (171). This can lead to the development of side toxicity which is a major concern for most of the nanoformulations. However, recent advances in nano-drug delivery have culminated the side toxicity by implementing the use of biphosphonates. In addition, combining NP formulation can modulate the tumor microenvironment to enhance the drug delivery. A combination of cycolopamine and paclitaxel nanoformulation designed to impede the Hh signaling disrupted the extracellular matrix of the tumor cells and increased drug profusion. In addition choice of nanoformulation, size of the NPs and their diffusion in the cell, cost of production, clinical translation, and cancer cell resistance are the limiting barriers that need to be addressed for designing specific Hh inhibitors. Overcoming these challenges can improve treatment methods for cancer patients. Altogether miRNAs and natural compounds mediated regulation of Hh signaling might help us devising new diagnostic/prognosis and therapeutics for CRC.

## Author Contributions

ZJ, MJ, and AR drafted the manuscript. HS, SR, AI, and WC revised the draft. HS, WK, WK-K, AG-L, JS-R, and WC conceptualized the study and reviewed it critically. All authors contributed to the article and approved the submitted version.

## Conflict of Interest

The authors declare that the research was conducted in the absence of any commercial or financial relationships that could be construed as a potential conflict of interest.
